# Effectiveness of motion-graphic video for informed consent in patients undergoing platelet-rich plasma therapy for androgenetic alopecia: a randomized controlled study

**DOI:** 10.3389/fdgth.2025.1713274

**Published:** 2026-01-12

**Authors:** Dichitchai Mettarikanon, Chime Eden, Patsaraporn Manunyanon, Veerayut Boonpit, Naparat Chookerd, Weeratian Tawanwongsri

**Affiliations:** 1 Division of Digital Content and Media, School of Informatics, Walailak University, Nakhon Si Thammarat, Thailand; 2 Informatics Innovation Center of Excellence, School of Informatics, Walailak University, Nakhon Si Thammarat, Thailand; 3 Division of Dermatology, Jigme Dorji Wangchuck National Referral Hospital (JDWNRH), Thimphu, Bhutan; 4 School of Informatics, Walailak University, Nakhon Si Thammarat, Thailand; 5 Division of Dermatology, Department of Internal Medicine, School of Medicine, Walailak University, Nakhon Si Thammarat, Thailand

**Keywords:** androgenetic alopecia, audiovisual education, digital health, informed consent process, motion graphic video, patient comprehension, patient satisfaction, platelet-rich plasma therapy

## Abstract

**Background:**

Audiovisual tools are increasingly used in healthcare to improve patient education and engagement. However, few studies, particularly in dermatology, have evaluated their effectiveness in enhancing patient understanding during the informed consent process. This study aimed to compare the effectiveness of a motion-graphic educational video with conventional verbal consent for patients undergoing platelet-rich plasma (PRP) therapy for androgenetic alopecia (AGA).

**Methods:**

In this randomized controlled trial, participants aged 18–55 years with AGA were recruited at the Dermatology Clinic, Walailak University Hospital, between December 2024 and March 2025. Participants were randomized to receive informed consent through either an educational video (Group A) or a conventional verbal explanation (Group B). Pre- and post-intervention knowledge and anxiety levels were assessed, and satisfaction was evaluated in Group A.

**Results:**

Thirty-four participants completed the study (73.5% male; median age: 39.5 years, IQR: 23.0). Median baseline knowledge and anxiety scores were 0.0 (IQR: 2.0) and 6.0 (IQR: 3.0), respectively. Post-intervention knowledge scores increased significantly in both groups (Group A: 9.0, IQR: 1.0; Group B: 7.0, IQR: 2.0; *p* < 0.001), with a greater knowledge gain in Group A (8.0, IQR: 3.0) compared to Group B (6.0, IQR: 2.0; *p* = 0.009). Anxiety scores remained unchanged in both groups. Group A reported a high usefulness score for the video (median, 10.0; IQR, 1.0). No significant correlations were found between demographic factors and baseline knowledge or anxiety.

**Conclusions:**

The motion-graphic educational video improved patient knowledge compared with conventional verbal explanations, without reducing anxiety. Participants reported high satisfaction, supporting the use of audiovisual media as an effective adjunct to the informed consent process.

**Clinical Trial Registration:**

https://www.thaiclinicaltrials.org/show/TCTR20241222004, identifier TCTR20241222004.

## 1 Introduction

Androgenetic alopecia (AGA) is a common form of hair loss with global prevalence rates ranging from 3.7% to 80% (1), being more prevalent among men than women (2). The condition is strongly age-dependent; by the age of 80 years, AGA affects approximately 73% of men and 57% of women (3). Treatment modalities for AGA include pharmacological and procedural approaches. The pharmacological treatments included topical minoxidil, oral finasteride, oral dutasteride, and low-dose minoxidil. Procedural treatments include hair transplantation, low-level laser therapy, microneedling, and autologous platelet-rich plasma (PRP) therapy (4). PRP therapy, a treatment gaining interest for hair restoration, involves the injection of plasma enriched with concentrated autologous platelets and growth factors, such as epidermal growth factor, insulin-like growth factor 1, and vascular endothelial growth factor, which are essential for hair follicle growth and regeneration (5, 6). This procedure involves multiple complex steps, including preparation methods, injection site preparation, injection techniques, and determination of the frequency and interval of injections (7, 8).

Despite the complex procedures involved, obtaining informed consent is crucial. Informed consent is a fundamental ethical principle that respects patient autonomy by providing clear, comprehensive information about procedures, empowering individuals to make well-informed healthcare decisions (9, 10). To obtain informed consent for medical procedures, it is crucial to address four key elements: general knowledge of the procedure (including indications, contraindications, steps, and post-procedure self-care), benefits, risks, and alternative treatments (11). It is also important to ensure that patients understand, retain, and process this information (12, 13). In addition to the traditional method of verbal explanations, which involves direct communication between healthcare providers and patients, the use of multimedia tools, such as videos, is increasingly prevalent and has demonstrated significant potential for improving the informed consent process in various medical settings (14). Multimedia tools enhance the informed consent process by improving patient knowledge, reducing anxiety, and increasing satisfaction through standardized and comprehensive information (15). They streamlined the process, allowing healthcare providers to focus on other tasks, while ensuring consistency (16).

Educational videos have been used in the informed consent process for various procedures; however, their use in dermatology remains limited (17). A previous study (18) conducted a randomized controlled study in the United States to evaluate whether video-based education delivered via mobile devices enhanced patient knowledge and satisfaction regarding informed consent and postoperative care for skin biopsies, compared with standard verbal instructions. The study included 84 participants. The video group showed a significant improvement in knowledge scores, whereas the control group did not. Both groups reported high levels of satisfaction. Another previous study (19) evaluated the efficacy of educational videos in improving patient understanding and reducing preprocedural anxiety in skin biopsies in a multicenter randomized controlled trial in Thailand. Fifty-four participants were included in the final analysis. Knowledge scores improved significantly in both groups after the intervention, with higher scores observed in the video group vs. the verbal informed consent group; however, this difference was not significant. Anxiety scores also decreased significantly in both groups, with no significant intergroup differences. The participants in the video group reported high levels of satisfaction and found the videos highly useful. For cosmetic procedures, patients often rely on the Internet as a primary source of information; however, the accuracy and validity of some online materials can be questionable. Few studies have examined patient comprehension, anxiety, and satisfaction during the informed consent process (20). PRP therapy for AGA is considered a cosmetic procedure; however, comprehensive informed consent interventions for this treatment are limited. To address this, we developed a motion graphic video for PRP therapy incorporating four key elements of informed consent grounded in evidence-based medicine. This study aimed to evaluate the effectiveness of this educational video compared with conventional verbal informed consent in patients undergoing PRP therapy for AGA.

## 2 Methods

### 2.1 Study design and participants

**Figure 1:**
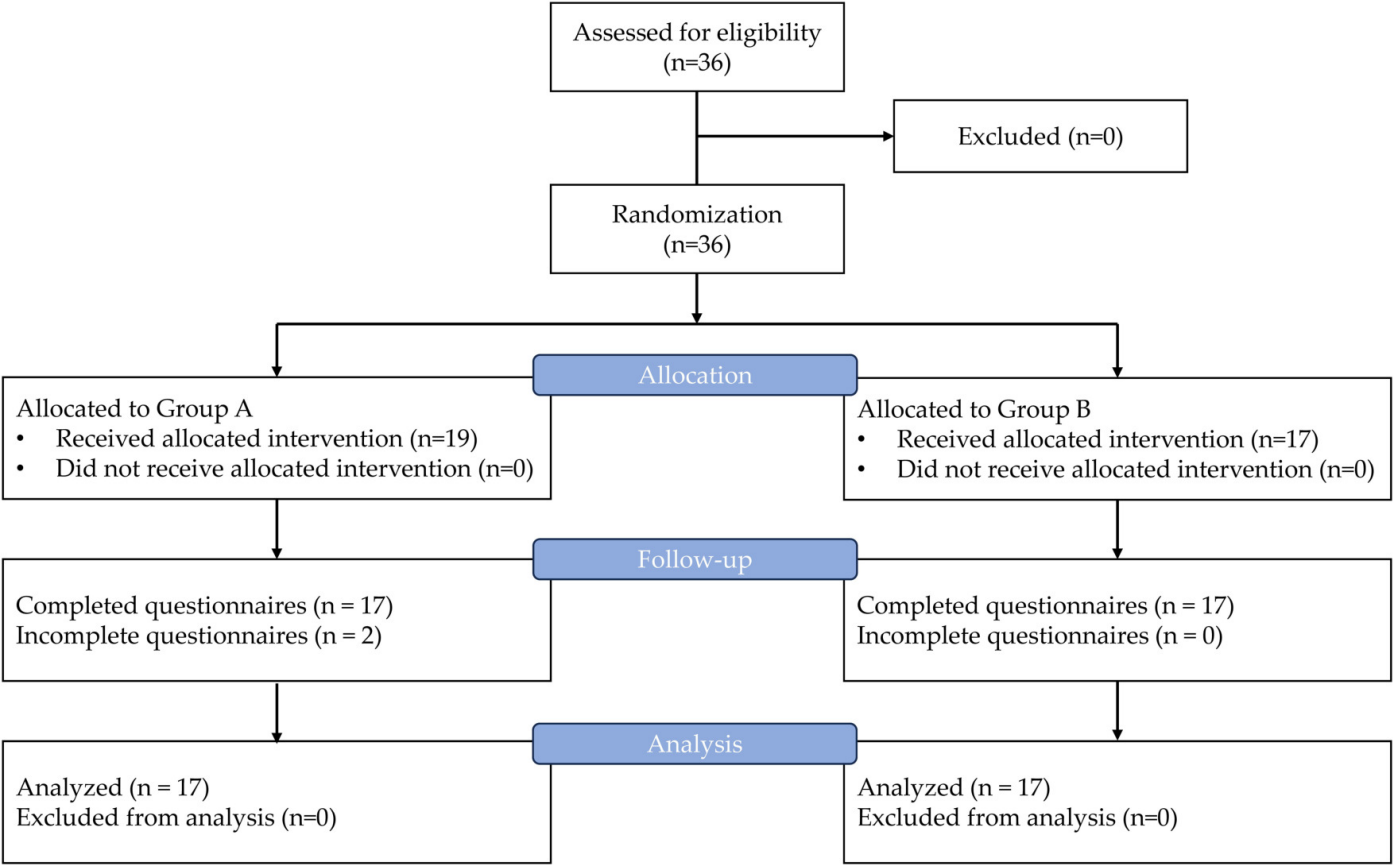
Study flow diagram detailing recruitment, allocation, intervention, and follow-up.

This randomized controlled trial was conducted at the Dermatology Clinic of Walailak University Hospital between December 2024 and March 2025. Participants were recruited by an independent researcher (V.B.) who was not involved in the clinical management to ensure that the process was free from coercion or undue influence. Individuals aged 18–55 years were eligible to participate. The severity of AGA in the male and female participants was rated as grades III–V on the modified Hamilton–Norwood scale and grades I–V on the Ludwig scale, respectively (21). The flowchart of the study is shown in Figure 1. Participants were excluded if they were unwilling to provide information, unavailable during the data collection period, or declined to participate. After recruitment, the participants were randomly assigned to one of two groups using simple randomization in Microsoft Excel: Group A (informed consent with a motion graphic video) or Group B (conventional informed consent with verbal explanation). Group A participants viewed a motion graphic video on a 12.9-inch tablet via a YouTube link (https://www.youtube.com/watch?v=Qp0JE07keIQ) and watched it once, whereas participants in Group B received a conventional verbal explanation from a dermatologist (W.T.). The duration of the verbal explanation in Group B was recorded. Allocation concealment was ensured using sequentially numbered, opaque, sealed envelopes. An independent researcher, not involved with recruitment, intervention delivery, or outcome assessment, developed and managed an envelope system (N.C.). Another researcher (V.B.), who was also not involved with the informed consent intervention nor a healthcare provider, was responsible for assessment of outcomes. V.B. distributed the pre- and post-intervention questionnaires and collected participants' self-reported anxiety outcomes using the State–Trait Anxiety Inventory (STAI). Baseline data including sex, age, educational level, comorbidities, Fitzpatrick skin type, and AGA severity scores (using the modified Hamilton–Norwood scale and Ludwig scale for the male and female participants, respectively) were collected. Pre- and postintervention knowledge and anxiety levels were assessed using validated questionnaires. Anxiety levels were measured using the State-Trait Anxiety Inventory (STAI), a five-item self-report measure of anxiety scored on a 4-point Likert scale (1–4 points per item) (22). Assessments were conducted before and immediately after the informed consent process in both groups. Additionally, satisfaction with the motion graphic video was evaluated in Group A using a scale ranging from 1 (extremely poor) to 5 (extremely satisfactory). Post-intervention, both groups attended a session to ask questions and clarify any misunderstandings, as per their usual practice.

### 2.2 Development of the educational video

Motion graphic videos use a combination of graphic design and animation to deliver information, narrate stories, and evoke emotional responses through dynamic visual communication (37). A 4-min, 50-s educational video was collaboratively developed by a multidisciplinary team. Two dermatologists (W.T. and C.E.) drafted the video content, focusing on accuracy and relevance. The content included details on the prevalence of hereditary hair loss, characteristics of PRP treatment, mechanism of PRP, treatment efficacy and indications, precautions, procedural steps, potential adverse effects, and post-treatment self-care in accordance with medical standards for informed consent prior to treatment. A motion graphic video development expert (D. M.) handled the motion graphics, including storyboarding, design elements, animation, and audio integration. The preparation of PRP and its step-by-step procedure were recorded, edited, and seamlessly merged with motion graphics provided by the media editor (P.M.). The final version of the video was thoroughly reviewed by the team (W. T., D. M., and P. M.) to ensure that it effectively communicated key information essential for the informed consent process.

### 2.3 Knowledge test development

Currently, no standard knowledge test is available to assess the patients' understanding of PRP treatment for AGA. The lack of a standardized tool makes it challenging to ensure consistent patient education and comprehension. To address this issue, we developed and validated a knowledge test with the help of two dermatologists (W.T. and C.E.) to evaluate participants' understanding before they underwent PRP treatment. The test is structured in a true-or-false format and consists of 10 items, each offering three response options: “true,” “false,” and “not sure.” We chose this format to simplify the assessment process and reduce the burden on the participants, allowing us to quickly gauge their understanding. The test was piloted on 30 participants from a dermatology clinic. Following the pilot study, we analyzed the index of item objective congruence (IOC) and included only items with an IOC value >0.5. Additionally, we calculated Cronbach's alpha to assess internal consistency, which yielded a value of 0.82, indicating acceptable reliability (38). Participants received a score of 1 for each correct answer, and a score of 0 for incorrect answers or responses marked as “not sure.” The knowledge test results are presented in Supplementary Table S1.

### 2.4 Sample size calculation

The sample size calculation determined that 34 participants were required for the study, who were divided equally into two groups of 17 participants each. The following formula was used to compare two independent means:

$$n=\frac{2\;\times {\left(Z_{\alpha /2}+Z_{\beta }\right)}^{2}\;\times \;\sigma^{2}}{\Delta^{2}}$$

This was based on the findings of Armstrong et al. (18). The parameters were set as follows: *α* error = 0.05, power = 0.80, standard deviation (SD) of the outcome variable (*σ*) = 1.5, minimum detectable difference (Δ) = 1.5, and an allocation ratio of 1:1. The initial calculation yielded 15 participants per group, and the final sample size was adjusted to 34 participants to account for an estimated 15% loss to follow-up.

### 2.5 Statistical analysis

Descriptive statistics are reported as frequency (percentage) for categorical variables and as median [interquartile range (IQR)] for continuous variables; the duration of verbal explanation is reported as mean [standard deviation (SD)]. Between-group comparisons were performed using the Wilcoxon rank-sum test for continuous variables and Fisher's exact test for categorical variables. Paired within-group comparisons were conducted using the Wilcoxon signed-rank test. Correlations were assessed using Spearman's rank correlation for ordinal–continuous associations and Pearson's correlation for relationships between continuous variables. All analyses were two-sided, and a *p*-value <0.05 was considered statistically significant. Statistical analyses were performed using R (version 4.3.2).

### 2.6 Approval by the ethics committee and clinical trial registration

This study was approved by the Walailak University Ethics Committee (WUEC-24-351-01). All participants provided written informed consent after receiving a comprehensive briefing about the study. The research adhered to the principles of the Declaration of Helsinki and the International Conference on Harmonization's Good Clinical Practice guidelines. It was registered with the Thai Clinical Trials Registry under the Foundation for Human Research Promotion in Thailand, ID TCTR20241222004.

## 3 Results

Overall, 36 participants were recruited for the study; however, 2 individuals from Group A were excluded owing to incomplete questionnaires, leaving 34 participants for the final analysis. The overall median age of participants was 39.5 years (IQR 23.0), while there was a predominance of male participants (25, 73.5%). Baseline characteristics, as shown in Table 1, were comparable for most variables; however, acne prevalence was higher in Group A (*p* = 0.039). No significant differences existed between the two groups with respect to metabolic disease or any of the other comorbidities. The participants included lecturers/professors (*n* = 6, 17.6%); students (*n* = 6, 17.6%); academics, officers, or civil servants (*n* = 16, 47.1%); freelancers (*n* = 1, 2.9%); engineers (*n* = 1, 2.9%); homemakers (*n* = 1, 2.9%); and businesspeople (*n* = 1, 2.9%). The motion-graphic video duration was 4 min 50 s, whereas the mean duration for verbal explanation in Group B was 4.74 min (SD 0.35).

**Table 1:** Baseline characteristics (*n* = 34).

Characteristic	Group A	Group B	*p* value
Age (years), median (IQR)	42.0 (15.0)	38.0 (24.0)	0.704
Male, *n* (%)	11 (64.7)	14 (82.4)	0.438
Fitzpatrick skin type, *n* (%)			0.497
Type II	5 (29.4)	2 (11.8)	
Type III	9 (52.9)	12 (70.6)	
Type IV	3 (17.6)	3 (17.6)	
AGA severity (male), *n* (%)			0.282
Grade III	10 (90.9)	10 (71.4)	
Grade IV	0 (0.0)	3 (21.4)	
Grade V	1 (9.1)	1 (7.1)	
AGA severity (female), *n* (%)			0.643
Grade I	2 (33.3)	1 (33.3)	
Grade II	4 (66.7)	1 (33.3)	
Grade III	0 (0.0)	1 (33.3)	
Comorbidity, *n* (%)			
No comorbidity	6 (35.3)	9 (52.9)	0.491
Acne	7 (41.2)	1 (5.9)	0.039
Metabolic diseases^a^	4 (23.5)	5 (29.4)	1.000
Others^b^	3 (17.6)	1 (5.9)	0.601
Educational level, *n* (%)			1.000
Below bachelor's degree	3 (17.6)	3 (17.6)	
Bachelor's degree	10 (58.8)	9 (52.9)	
Master's degree	1 (5.9)	1 (5.9)	
Doctoral degree or higher	3 (17.6)	4 (23.5)	

AGA, androgenetic alopecia; IQR, interquartile range.

a Metabolic diseases include essential hypertension, dyslipidemia, and type II diabetes mellitus.

b Others include major depressive disorder in a controlled state, chronic low back pain, chronic hepatitis B infection, and allergic rhinitis.

At baseline, the median knowledge score of participants was 0.0 (IQR 2.0) and anxiety score was 6.0 (IQR 3.0). Before the intervention, the median knowledge score of the participants did not significantly differ between Group A (0.0, IQR 2.0) and B (1.0, IQR 2.0; *p* = 0.955). Similarly, the median anxiety score of the participants did not significantly differ between Group A (5.0, IQR 3.0) and B (7.0, IQR 3.0; *p* = 0.882). In Group A, post-intervention knowledge scores (median 9.0, IQR 1.0) were significantly higher than pre-intervention scores (median: 0.0, IQR 2.0; *p* < 0.001). However, the pre-intervention (median 5.0, IQR 3.0) and post-intervention (median 5.0, IQR 3.0; *p* = 0.357) anxiety scores were not significantly different. In Group B, the post-intervention knowledge scores (median 7.0, IQR 2.0) were significantly higher than the pre-intervention knowledge scores (median 1.0, IQR 2.0; *p* < 0.001). In contrast, the pre-intervention (median 7.0, IQR 3.0) and post-intervention (median 5.0, IQR 3.0; *p* = 0.395) anxiety scores showed no significant differences.

**Figure 2:**
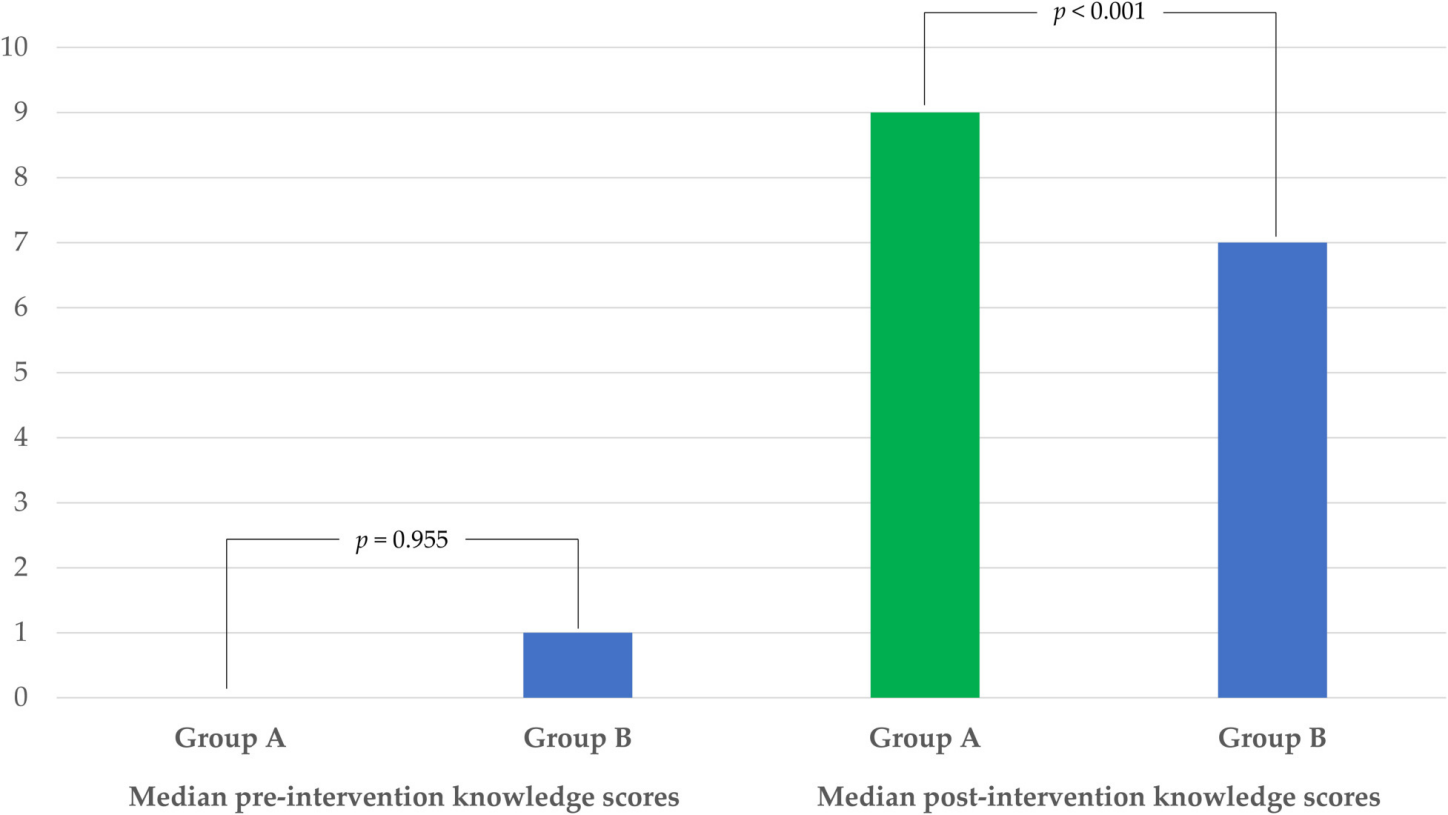
Comparison of median pre- and post-intervention knowledge scores between group A and B.

After the intervention, Group A participants had a significantly higher median knowledge score (9.0, IQR 1.0) than did Group B participants (7.0, IQR 2.0; *p* < 0.001), as illustrated in Figure 2. The median anxiety scores did not significantly differ between Group A (5.0, IQR 3.0) and B (5.0, IQR 3.0; *p* = 0.892). The increase in the median knowledge scores was also significantly greater in Group A (8.0, IQR 3.0) than in Group B (6.0, IQR 2.0; *p* = 0.009). Conversely, no difference was observed in the change in anxiety scores between Group A (0.0, IQR 0.0) and B (0.0, IQR 0.0; *p* = 0.973). Group A participants rated the educational media highly, with a median usefulness score of 10.0 out of 10.0 (IQR 1.0).

The median preintervention knowledge scores did not significantly differ between male (1.0, IQR: 2.0) and female (0.0, IQR 1.0; *p* = 0.242) participants. Similarly, the median preintervention anxiety scores did not differ significantly between male (5.0, IQR 3.0) and female (7.0, IQR 3.0; *p* = 0.722) participants. Pearson's correlation analysis revealed no significant relationship between age and preintervention knowledge (*r* = −0.050, *p* = 0.777) or preintervention anxiety scores (*r* = 0.062, *p* = 0.728). Spearman's analysis also showed no significant correlation between educational level and preintervention knowledge scores (*ρ* = −0.045, *p* = 0.801) or preintervention anxiety scores (*ρ* = −0.047, *p* = 0.793). After the intervention, Group A participants were asked to rate their perspectives on educational media. They reported high levels of satisfaction across all aspects. Table 2 summarizes their perspectives on the motion graphic videos.

**Table 2:** Participants’ satisfaction scores for the motion graphic video (*n* = 17).

Aspects	Median (IQR)
The motion graphic video was clear and easy to follow.	5.0 (1.0)
The narrative progressed in a logical order.	5.0 (1.0)
The content was relevant and appropriate.	5.0 (1.0)
The visuals matched and supported the presented information.	5.0 (1.0)
The illustrations were clear, easy to interpret, and visually engaging.	5.0 (1.0)
The background music complemented the content.	5.0 (1.0)
The narration was clear, with proper volume and tone.	4.0 (1.0)
The length of the media clip was appropriate.	5.0 (1.0)
The subtitles were clear and helpful.	5.0 (1.0)

IQR, interquartile range.

## 4 Discussion

Ensuring that participants are fully informed before consenting to medical procedures is an ethical and legal obligation. The informed consent process is based on core ethical principles including patient autonomy, beneficence, and nonmaleficence (23). However, reportedly, only one-third of participants achieve a satisfactory level of understanding, highlighting a significant gap between the intent of informed consent and patients' comprehension (24). In addition to conventional verbal explanations, digital technology has been shown to improve understanding without negatively affecting satisfaction or anxiety (25). Our findings are consistent with these observations, demonstrating that motion graphic videos effectively enhance knowledge before procedures without adversely affecting anxiety levels. The participants reported high satisfaction, emphasizing its potential as a valuable tool for improving patient understanding.

Several interventions, including written materials, audiovisual content, and multicomponent approaches, have been proven to improve patients' comprehension of informed consent processes for various medical and surgical procedures. Educational videos are among the most effective interventions utilized in the informed consent process for a range of procedures; however, their application in dermatology remains limited (17). These findings are consistent with those of previous studies. A previous study (18) conducted a randomized controlled trial with 84 patients undergoing skin biopsies and compared the effectiveness of conventional verbal education with that of educational videos delivered via portable devices. The video group experienced a significant improvement in knowledge scores (mean ± SD: 1.55 ± 1.71), whereas the verbal education group did not show significant gains (mean ± SD: 1.12 ± 1.74). Despite the lack of a significant intergroup difference in knowledge improvement, participants in the video group rated the educational content favorably (mean ± SD: 9.01 ± 1.5 for usefulness and 9.01 ± 1.66 for appeal). Both groups expressed high levels of satisfaction with the educational methods. Another previous study (19) carried out a multicenter randomized controlled trial to evaluate the effectiveness of educational videos compared with that of conventional verbal explanations in 54 participants undergoing skin biopsies. Both approaches significantly enhanced post-intervention knowledge and reduced anxiety. However, although the video group achieved a greater median improvement in knowledge scores (2.5; IQR: 1.0–5.0), compared with the verbal group (1.5; IQR: 0.0–4.0), the difference was not significant. The reduction in anxiety levels was similar in both groups. Participants in the video group expressed high levels of satisfaction with the educational material.

Audiovisual media surpass verbal explanations in enhancing understanding because of several key factors. First, audiovisual media simultaneously engage multiple senses, promoting improved comprehension and retention of information. The integration of visual and auditory elements reduces cognitive load, enabling learners to process and understand complex concepts more effectively (26, 27). Second, visual aids are particularly advantageous for visual learners, who benefit from seeing the material they are learning. The reinforcement provided by visual images can bridge the gap between vague awareness and a clear and lasting understanding of concept (28). Third, audiovisual media can evoke emotional responses essential for deeper engagement and better retention. It encourages active participation rather than passive consumption (29).

One possible explanation for the effectiveness of our audiovisual intervention is that it was collaboratively developed and validated by board-certified dermatologists and experts in media development. This collaboration ensured that the content was accurate and engaging, and presented information in an attractive and easily digestible manner. The credibility and quality of the content found on social media platforms are often questionable because of a lack of professional oversight. Without dedicated gatekeepers monitoring the accuracy of the information, false or misleading content can spread rapidly, sometimes with significant real-world consequences (30). Even content with inaccurate information can appear credible owing to positive feedback or a high user reputation, further complicating the efforts to identify reliable sources (31). Misinformation poses a significant threat to public health. Studies have shown that false information sources often provoke greater outrage than do trustworthy sources, leading users to impulsively reshare such content, often without verifying its accuracy (32). This finding highlights the critical role of medical providers in developing accurate and well-designed educational tools to ensure that patients receive reliable information. Such efforts not only help prevent misunderstandings but also reduce unnecessary anxiety, fostering more confident and well-informed patients.

Research has consistently highlighted the effectiveness of audiovisual interventions in reducing anxiety levels, often outperforming verbal explanations. One study (33) conducted a randomized study with 94 pregnant women scheduled for amniocentesis who were divided into two groups: one received information through a video presentation and the other received verbal explanations. Although both methods significantly reduced anxiety, the video group achieved greater reductions in the STAI-I (*p* = 0.0001) and Amsterdam Preoperative Anxiety and Information Scale scores (*p* = 0.001). Women in the video group requested significantly less additional information (*p* = 0.0001), whereas no significant reduction in requests was observed in the verbal group (*p* = 0.654). Another study (34) explored anxiety reduction among parents of children scheduled for hypospadias surgery. Participants were assigned to one of two groups: one receiving structured audiovisual education or another provided with traditional verbal explanations. Although both approaches reduced anxiety levels after the second consultation, the audiovisual group exhibited significantly lower STAI-I scores than did the verbal information group (*p* = 0.001). This finding underscores the effectiveness of structured audiovisual education in mitigating preoperative anxiety among parents. The other study (35) conducted a prospective study to evaluate anxiety levels in patients undergoing their first computed tomography scan using the STAI. Both written materials and audiovisual interventions reduced state anxiety compared with baseline levels (*p* < 0.001). However, audiovisual resources proved more effective, compared with written materials (mean anxiety score: 38.6 ± 7.7 vs. 43.2 ± 5.5). The trait anxiety scores remained unaffected (*p* = 0.31). These results highlight the significant role of audiovisual materials in reducing anxiety before medical procedures. However, our findings showed no significant difference in anxiety scores between Group A and Group B after the intervention. It is possible that these could be due to low anxiety levels measured before treatment [median 6.0 (IQR 3.0)], which is below the clinical anxiety threshold; thus, there was less possibility of decreasing anxiety even more, also known as a floor effect (36). Another reason could be that the procedure required multiple injections, which might have caused at least a slight increase in anxiety among participants. However, it was still classified as minimally invasive, as the level of discomfort was not substantial enough to result in a greater rise in anxiety compared to what the educational method could offer. A smaller sample size may have limited our ability to detect small differences in anxiety between the two groups. Future studies with larger sample sizes in various types of clinical settings are needed to further assess the impact of audiovisual interventions on participants' anxiety level in a clinically relevant manner.

This study presents several limitations. First, it is difficult to generalize because it is a single-center study with a small sample size. A significant number of participants in the study exhibited minimal anxiety, which, coupled with a limited sample size, diminished the statistical power to discern minor reductions in anxiety levels across groups. Future studies should utilize larger sample sizes and conduct multiple-site randomized controlled trials to validate these findings and determine the efficacy of this intervention in different healthcare settings. Second, this study had the inability to blind participants and the clinician who provided informed consent. The design of the study was thus unblinded, which may potentially introduce bias into the performance and detection of the study; nonetheless, the outcomes were assessed using standardized questionnaires, and an independent investigator who was not involved in the informed-consent process or clinical care administered and collected the questionnaires and scored the knowledge assessments using a predetermined scoring key, thereby reducing the risk of detection bias. Third, the predominance of highly educated individuals within the sample, as indicated by a large proportion of participants with at least a bachelor's degree. This may limit the generalizability of the study findings to individuals with lower health literacy or lower educational attainment, in whom the effectiveness of motion-graphic–based informed consent may differ. Future studies should expand the range of literacy levels represented in the study population and develop tailored motion graphic materials specifically for low-literacy audiences. Fourth, this study did not evaluate knowledge retention over an extended period of time. As a result, there is no way of knowing whether the benefits of the audiovisual informed-consent intervention were sustained after the original study concluded. Future studies should involve a longer follow-up interval to assess retention of knowledge and the long-term effect of the intervention. Finally, although the intervention resulted in improvement in knowledge scores immediately following the provision of informed consent, it is still unknown whether the improvement in knowledge level will result in clinically meaningful benefits to participants, as downstream metrics such as quality of decision-making, decisional conflict, and follow-up care or return to care behaviors were not evaluated. Therefore, improvements in knowledge levels should not be interpreted as a direct correlation with improved clinical outcomes. Future studies should evaluate how higher levels of knowledge correlate to improved decision-making and improved patient-centered outcomes, particularly appropriate follow-up care and timely returns for further medical consultation or intervention based on complications.

## 5 Conclusions

The educational video of motion graphics used in this study was found to improve the immediate comprehension of patients regarding PRP therapy for AGA compared to written informed consent given verbally but did not change patient anxiety levels. It was also perceived to be very beneficial in providing evidence for using audiovisual media to support verbal information as effective tools for providing patients with accurate and consistent information about a treatment option. Each patient still requires at least one session with an individual to address questions and concerns, ensuring that any unique issues are discussed. This is essential for confirming that patients fully understand PRP therapy before proceeding with the treatment. The limited sample size, single-center design, and elevated educational attainment of participants restrict the generalizability of the findings. Larger multicenter studies would be needed to confirm these findings, especially if future studies could evaluate long-term retention of knowledge and eventual effects on patient outcomes such as quality of decision-making related to the procedure and self-management post-procedure.

## Data Availability

The original contributions presented in the study are included in the article/Supplementary Material, further inquiries can be directed to the corresponding author.
