# Supplementary material for: Effectiveness of motion-graphic video for informed consent in patients undergoing platelet-rich plasma therapy for androgenetic alopecia: a randomized controlled study
Source: Front Digit Health. 2026 Jan 12;7:1713274. doi: 10.3389/fdgth.2025.1713274 (PMC12832857; doi:10.3389/fdgth.2025.1713274)
Supplement: Supplementary file 1 [file Table1.docx]

**Supplementary Table 1**. Knowledge test about platelet-rich plasma therapy for androgenetic alopecia

| **Item** | **True** | **False** | **Not sure** |
| --- | --- | --- | --- |
| 1. The treatment of androgenetic alopecia with platelet-rich plasma is suitable for patients at all levels of disease severity. |  |  |  |
| 1. The treatment of androgenetic alopecia with platelet-rich plasma should be avoided in pregnant women due to limited available research data. |  |  |  |
| 1. The procedure requires drawing 15 to 20 milliliters of blood from the patient per treatment session. |  |  |  |
| 1. Patients may receive topical or injectable anesthetics on the scalp before the procedure to reduce pain during the treatment. |  |  |  |
| 1. After blood is drawn, the physician will centrifuge the blood to separate the plasma, which takes approximately 10 minutes. |  |  |  |
| 1. Before the procedure, the patient's scalp will be disinfected with an antiseptic solution. |  |  |  |
| 1. The platelet-rich plasma injection procedure uses a needle similar in size to a blood draw needle and is administered to the scalp. |  |  |  |
| 1. The treatment increases hair count by approximately 28% and hair thickness by about 32%. |  |  |  |
| 1. The treatment of androgenetic alopecia with platelet-rich plasma has no severe side effects. |  |  |  |
| 1. The treatment of androgenetic alopecia with platelet-rich plasma may cause side effects such as headache, pain during the procedure, and temporary swelling or redness. |  |  |  |
